# Monitoring the Conformational Changes of the Aβ(25−35) Peptide in SDS Micelles: A Matter of Time

**DOI:** 10.3390/ijms24020971

**Published:** 2023-01-04

**Authors:** Angelo Santoro, Michela Buonocore, Manuela Grimaldi, Enza Napolitano, Anna Maria D’Ursi

**Affiliations:** 1 Department of Pharmacy, University of Salerno, Via Giovanni Paolo II, 132, 84084 Fisciano, Italy; 2 Department of Pharmacy, Scuola di Specializzazione in Farmacia Ospedaliera, University of Salerno, Via Giovanni Paolo II, 132, 84084 Fisciano, Italy; 3 Department of Veterinary Pathology, University of Naples Federico II, Via Federico Delpino 1, 80137 Naples, Italy; 4 PhD Program in Drug Discovery and Development, Department of Pharmacy, University of Salerno, 84084 Fisciano, Italy

**Keywords:** Alzheimer, Aβ(25−35), NMR, structural biology, micelles

## Abstract

Alzheimer’s disease is a neurodegenerative disease characterized by the formation of amyloid plaques constituted prevalently by amyloid peptides. Due to the well-known challenges related to the study in solution of these peptides, several membrane-mimicking systems such as micelle constituted by detergent—i.e., DPC and SDS—have been deeply investigated. Additionally, the strategy of studying short fragments instead of the full-length peptide turned out to be advantageous in exploring the structural properties of the different moieties in Aβ in order to reproduce its pathologic effects. Several studies reveal that among Aβ fragments, Aβ(25−35) is the shortest fragment able to reproduce the aggregation process. To enrich the structural data currently available, in the present work we decided to evaluate the conformational changes adopted by Aβ(25−35) in SDS combining CD and NMR spectroscopies at different times. From the solved structures, it emerges that Aβ(25−35) passes from an unordered conformation at the time of the constitution of the system to a more ordered and energetically favorable secondary structure at day 7, which is kept for 2 weeks. These preliminary data suggest that a relatively long time affects the kinetic in the aggregation process of Aβ(25−35) in a micellar system, favoring the stabilization and the formation of a soluble helix conformation.

## 1. Introduction

Alzheimer’s disease (AD) is a neurodegenerative disease responsible for the slow and progressive destruction of brain cells, a condition which promotes the onset of total mental decline [1,2,3]. Nowadays, over 50 million people are affected by Alzheimer’s or related dementia [4]. Based on the amyloid cascade hypothesis, the neurodegeneration caused by AD is due to the formation of fibrils composed of aggregated amyloid peptides and consequent plaques [5,6,7,8,9]. It is known that the aggregation process of amyloid-β (Aβ) peptides may be influenced by different factors, like metal ions, pH, temperature, and the environment in which they are located [10,11,12]. Several studies based on solid-state nuclear magnetic resonance (ssNMR) demonstrated that the full-length Aβ(1−40) and Aβ(1−42) tend to form polymorphic protofibrils which rearrange as raw β-sheet structures, predictive of β-organized superstructures in mature fibrils. However, the intermediate states leading to protofibrils are still under investigation [13,14,15]. Moreover, the rapid aggregation mechanism of these peptides raises an issue in setting the in vivo conditions to study Aβ peptides in solution because a barely aqueous system would drive the self-interaction between the highly hydrophobic C-terminal region and the central Aβ moiety, forming the transient β-hairpin crucial for the aggregation process [9,16].

Although Aβ peptides tend to fibrillate in plain water, the interaction with the cell membrane is believed to be crucial for the pathological role of the peptide [17]. Therefore, several membrane-mimicking systems have been explored to study Aβ and its fragments. Mixtures of organic solvents—such as trifluoroethanol (TFE) and 1,1,1,3,3,3-hexafluoro-2-propanol (HFIP)—with water were largely exploited mainly in early structural studies of Aβ peptides [18,19,20,21,22,23,24,25,26]. Micelles, on the other hand, represent one of the most used membrane-mimetic systems thanks to their low molecular weight and high reproducibility. In a recent study, Serra-Batiste et al. explored various surfactant micelles for the formation of oligomeric complexes. They demonstrated that in dodecyl phosphocholine (DPC) micelles Aβ(1−42) peptide, rather than Aβ(1−40), β-sheet-structured oligomers tends to form due to the higher hydrophobic nature of the longer amyloid-β fragment [27,28]. Another membrane-mimetic system extensively used for studying Aβ peptides is sodium dodecyl sulphate (SDS) micelles. Indeed, Aβ peptides are characterized by an overall positive charge, which allows the peptides to interact with the negatively charged surface formed by SDS micelles [21,29]. Conformational studies of wild-type and mutant Aβ peptides in SDS demonstrated that the peptide–micelles interaction is significantly affected by the primary structure [30,31,32]. Still, obtaining the full-length amyloid peptides is not a simple task, and several works have focused their study on shorter domains of Aβ peptide, which are excellent starting points for probing the behavior of parent proteins in different systems [24,33,34,35,36,37]. Several Aβ fragments react similarly to the parent peptides when placed in the SDS micelle system. In particular, different studies have been performed to investigate the behavior in SDS micelles of Aβ fragments encompassing residues ^10^Y-M^35^ [38,39,40,41]. Among these fragments, Aβ(25−35) represents the shortest sequence of Aβ able to mimic the biological behavior of the full-length amyloid peptides, forming large β-sheet aggregates and reproducing the toxicity of the peptide [21,42,43,44,45]. Conformational studies indicate that Aβ(25−35), like Aβ(1−42), undergoes a conformational transition depending on the environmental conditions, passing from a soluble and unordered secondary structure to an aggregated fibrillary β-sheet structure [46]. Previous conformational analysis of Aβ(25−35), performed with nuclear magnetic resonance (NMR) in SDS and LiDS micelle solutions, demonstrated the presence of a helix on ^28^K-L^34^, proving that Aβ(25−35) has structural features similar to its parent peptide Aβ(1−42) [47]. As the amyloid peptide tends to aggregate over time, the great threat is represented by the final formation of the pathological amyloid plaques [48,49,50]. Because this often represents a point of no return, it is fundamental to mitigate and, in the most promising hypothesis, reverse this process while in the prodrome stages of the pathology. Although it is assessed that the setting of the environment is crucial to modulate the conformational events that bring to the formation of the fibrils, little is known about how time would gradually affect the secondary structure of amyloid in solution. In this work, we exploited Aβ(25−35) as a model to mimic the structural features of the Aβ(1−42) full-length, in the folding–unfolding process, with careful attention to the conformational intermediates occurring during the soluble-aggregate transition. To this end, we performed circular dichroism (CD) and NMR analysis to evaluate the effect of SDS micelles on the conformation of Aβ(25−35) at days 0, 4, 7, and 14; additionally, we measured the diffusion coefficients and the hydrodynamic radii of Aβ(25−35) at different times to investigate the behavior of the peptide–micelle complex.

## 2. Results

### 2.1. Circular Dichroism Experiments

**Figure 1:**
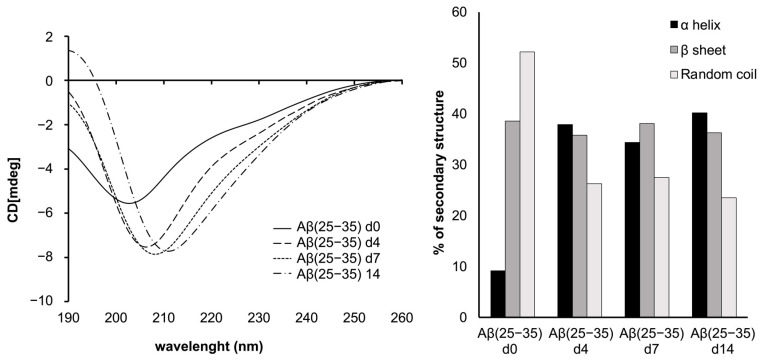
CD curves and secondary structure quantification performed with CONTIN algorithm of Aβ(25−35) peptide in SDS micelles at the time of the constitution of the system and after 4, 7 and 14 days.

Figure 1 shows CD spectra of Aβ(25−35) recorded in SDS micelle solution at the time of the constitution of the system and after 4, 7, and 14 days. The CONTIN analysis indicates that Aβ(25−35) in SDS micelles at day 0 presents 52% of random coil and 39% of β-sheet conformation. After 4 days the content of β-sheet is unchanged, but there is an increase in the helix conformation (35–40%) at expenses of the random coil conformation. The increased ratio in helix conformation is conserved for the full duration of the experiments.

### 2.2. NMR Spectroscopy

#### 2.2.1. DOSY Experiments

To analyze the diffusion behavior of Aβ(25−35) peptide in the SDS micelle solution over time, we recorded pseudo-2D DOSY experiments. Details about DOSY spectra and diffusion curves are reported in Figures S1 and S2. Table 1 shows the diffusion coefficients of SDS micelles and Aβ(25−35) peptide, respectively.

The diffusion coefficients (D) calculated from DOSY spectra for SDS micelles and Aβ(25−35) at different time points are very similar. The calculation of the hydrodynamic radius are based on the diffusion coefficient of Aβ(25−35) and SDS detergent, respectively, using 1,4-dioxane as a reference resulted in a 26 Å hydrodynamic radius [51]. This value corresponds to the R_h_ calculated for SDS micelles in water [52,53,54]. It is constant for all the experimental conditions, and as is common to SDS and Aβ(25−35) peptide, indicates an interaction of the peptide with the SDS micelles which is conserved over time. 

#### 2.2.2. Analysis of Aβ(25−35) Structures

1D ^1^H, 2D ^1^H-^1^H Total Correlation Spectroscopy (TOCSY) and Nuclear Overhauser Effect Spectroscopy (NOESY) spectra of Aβ(25−35) in SDS micelles at 0, 4, 7 and 14 days were collected on a Bruker 600 MHz at 298 K (Figures S3–S6). A ^1^H chemical shift assignment was carried out by iteratively analyzing TOCSY and NOESY spectra with SPARKY [55,56]. 2D ^1^H-^1^H spectra show 11 well-dispersed amide chemical shifts and uniform resonance line widths according to the characteristics of a structured peptide (Tables S1–S4). The sequential chemical shift assignment was performed according to the Wüthrich procedure [57]. The NOEs were translated into interprotonic distances using CALIBA routine of CYANA 3.1 software and then used as restraints for the NMR structure calculations [58]. Table 2 reports the statistics for the structural calculation of the NMR ensemble of Aβ(25−35) peptide at 0, 4, 7, and 14 days in SDS micelles. The table shows a significant increase in total NOEs recorded in the different NOESY spectra over time.

**Figure 2:**
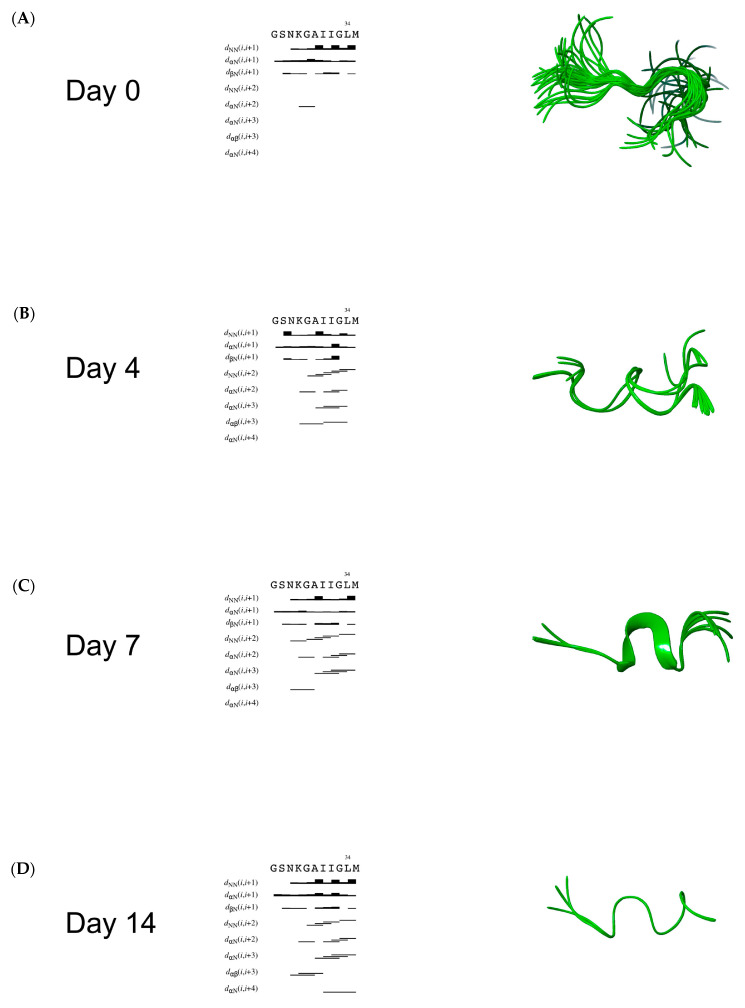
On the left, overview of the sequential and medium-range nuclear Overhauser enhancements (NOEs) used to calculate the Aβ(25−35) structure ensembles obtained at day 0 (**A**), day 4 (**B**), day 7 (**C**) and day 14 (**D**). On the right, ribbon visualization of the representative structures of the corresponding calculated ensembles.

Figure 2 summarizes the sequential and medium-range NOE effects observed in the 2D NOESY spectra. The sequential NOE plots report at day 0 only one α,N(i,i+2) effect between residues ^29^G-G^33^. The paucity of NOE reveals the prevalence of disordered conformations with the presence of rare half-turn structures in the central part of the peptide. From day 4, several N,N(i,i+2), α,N(i,i+2), α,N(i,i+3) and α,β(i,i+3) effects indicate the rising of turn-helical structures involving the residues ^29^G-M^35^. On days 7–14, additional NOEs are observable in the N-terminal region, consistent with the rising of stable, regular secondary structures including all the peptide sequence. Interestingly, analysis of the NMR structure bundle indicates a progressive reduction of the conformer families moving from day 0 to day 14. At the beginning, Aβ(25−35) is disordered: a variety of conformer populations are evident, with a sporadic half-turn on the N-terminus. From day 4, high occurrence of regular conformations is evident, with the definition of a 3_10_ helix on the residues ^28^K-I^32^ at day 14.

**Figure 3:**
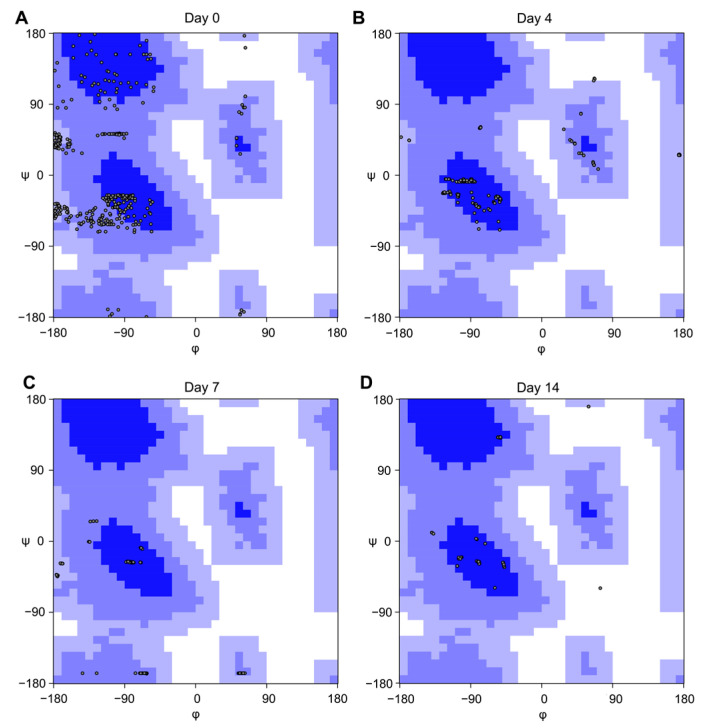
Ramachandran plot of Aβ(25−35) peptide at (**A**) day 0, (**B**) day 4, (**C**) day 7, and (**D**) day 14 in SDS micelles.

The Ramachandran plots in Figure 3 confirm that Aβ(25−35) at day 0 is characterized by three different clusters of conformations, which are β-sheet, right-handed and left-handed helix. Starting from day 4, the peptide loses the contribution provided by the β-sheet secondary structure, still conserving both orientations of the helix conformation. Conversely, at days 7 and 14, the peptide assumes predominantly right-handed helix conformation. 

Procheck-NMR analysis performed on the solved Aβ(25−35) PDB structures [59] allowed obtaining the Ramachandran plot for each residue of the NMR-calculated bundle of structures. Based on this analysis, we observed that Aβ(25−35) N-terminal and C-terminal residues tend to assume over time dihedral angle values close to those of a right-handed helix (Figure S9A–D). By comparing these values with those deposited in PDB for Aβ(1−40) NMR structure in SDS (PDB ID: 1BA4) (Figure S9E), it is possible to affirm that the structure of the short Aβ(25−35) after 14 days is similar to the Aβ(1−40)’s, validating the use of Aβ(25−35) as a valuable Aβ(1−40) structural model [29].

## 3. Discussion

Aggregation of Aβ peptide is a matter of time and modulating the formation of the monomers or the soluble fibrils could represent a winning strategy to prevent AD [60]. Unfortunately, this is a very difficult task because of the tendency of amyloid peptides to aggregate in aqueous conditions, which makes these molecules troublesome to study in an experimental context. In this regard, systems of micelles composed of SDS have been exploited to study the solution structures for the full-length Aβ(1−42) and several fragments [27,28,30,31,61], among which, Aβ(25−35) represents the shortest portion capable of mimicking the aggregation process [21,24,42,45,47]. In this work, we study the behavior of Aβ(25−35) in SDS at 0, 4, 7, and 14 days to gain insights on the conditions in which this fragment can reproduce to the greatest extent the features of the full-length in this system. Preliminary CD analysis shows that Aβ(25−35) in SDS passes from a tendentially disordered conformation at day 0, characterized by prevalent random coil and β-sheet conformations, to a more ordered one, after four days, where the helix conformations rise and increase for over the experimented time (Figure 1). Diffusion experiments performed by NMR spectroscopy evidence that the peptide Aβ(25−35) interacts with the micelles right from the early stages, suggesting a behavior comparable with other amyloid peptide fragments, whose interaction with SDS micelles has been widely studied [41,61]. This interaction is maintained throughout the analysis as confirmed by the diffusion values and the hydrodynamic radii similar to SDS micelles’ ones, in accordance with data reported in literature [62,63,64]. Two-dimensional TOCSY and NOESY experiments revealed that NOE effects between the peptide’s protons significantly increase from day 0 to day 4, particularly in the ^28^K-M^35^ region. Indeed, at days 7–14, this effect is also extended to residue ^27^N, suggesting that the peptide tends to stabilize its conformation over time. The transition to ordered conformations is evident in the 2D-NOESY spectra with the appearance of new inter-residues peaks. Aβ(25−35) in SDS passes from unstable β-like conformation to a more ordered and stable α-helix structure encompassing the residues ^29^G-M^35^ after 7 days (Figure 2). Eventually, at day 14, this α-helix conformation converts to a 3_10_ helix and shifts on the residues ^28^K-I^32^. These data suggest that the C-terminus represents the Aβ(25−35) moiety most affected by the effects of the time in the proposed system. The Ramachandran plot analysis supports this evidence: at day 0 Aβ(25−35) presents a structure with a dense cluster of dihedral angles in the β-sheet region (Figure 3A), which is already lost at day 4 (Figure 3B) in favor of a rather helical structure, whereas at days 7 and 14 there is a lower number of clusters, all concentrated in the region of right-handed helix dihedral angle values (Figure 3C,D). By analyzing the Ramachandran plot residue by residue, it is possible to observe that the residues primarily involved in the β-sheet conformation are situated in the N-terminus (Figure S9A). However, it is possible to observe that for all the residues of the sequence the dihedral angle values tend to cluster at day 7 in helix conformations, except for the ^33^G-L^34^ amino acids which are characterized by regular helix structures only at day 14. Remarkably, by comparing the Aβ(25−35) dihedral angle values obtained on the last day with those of the corresponding residues of Aβ(1−40) in SDS (PDB ID: 1BA4), it is possible to observe a significant similarity of the structures (Figure S9D,E). In conclusion, this explorative study highlights that the amyloid fragment may prefer a 7-day delay of settling from the time of the constitution of the system to assume energetically favorable conformations, similar to those of the parent Aβ(1−40) amyloid peptide in the same conditions. Therefore, it is mandatory that special attention be given to the choice of timing when negatively charged micelles are chosen for structural studies.

## 4. Materials and Methods

### 4.1. Sample Preparation

#### 4.1.1. Aβ(25−35) Peptide Synthesis

Aβ(25−35), was manually synthesized using Fmoc/tBu solid-phase peptide synthesis (SPPS) following Merrifield strategy [37,65]. The peptide was purified by reversed-phase chromatography (HPLC) using Phenomenex C18 column. The peptides were characterized on a Finningan LCQ Deca ion trap instrument equipped with an electrospray source (LCQ Deca Finnigan, San José. CA, USA). The samples were directly infused in the ESI source using a syringe pump at a flow rate of 5.0 mL/min. The data were analyzed using the Xcalibur software. The sample purity was >98%.

#### 4.1.2. Sample Preparation for Analyses

Before performing experiments, Aβ(25–35) peptide was previously treated according to the defibrillation procedure [66]. Subsequently, SDS micelles were prepared by dissolving Aβ(25–35) peptide in an SDS/PBS (pH 7.4) mixture. To obtain SDS micelles, we used a concentration of 80 mM, which is 10-fold the SDS critical micellar concentration (c.m.c.) [67]. The final concentration of Aβ(25–35) peptide was 0.15 mM. 

### 4.2. CD Experiments

CD spectra were obtained using a JASCO J-810 spectropolarimeter, with the aid of a 1 mm long quartz cell, working at a temperature of 25 °C. The CD curves were acquired by an average of 4 scans, in a measuring range of 260-190 nm, at a bandwidth of 1 nm and at a scanning speed of 10 nm/min. Each spectrum was processed by subtracting the solvent spectrum. The analysis of the CD curves was performed using the CONTIN algorithm of the online platform DICHROWEB [68,69]. 

### 4.3. NMR Experiments

#### 4.3.1. NMR Data Recording and Processing

Aβ(25−35) and SDS-d_25_ were prepared as described before. All NMR samples were given 10% (v/v) D_2_O. Further, 1D, 2D (^1^H-^1^H-TOCSY and ^1^H-^1^H-NOESY), and pseudo-2D (DOSY) experiments were recorded at 25 °C on a Bruker Avance 600 MHz spectrometer equipped with a 5 mm triple resonance ^1^H, ^13^C and ^15^N, z-axis pulsed-field gradient probe head. The water signal was suppressed using the excitation sculpting gradient pulse [70]. All the spectra were transformed and visualized in TopSpin 3.1 (Bruker Biospin). For the structure calculation of Aβ(25−35) peptide at different time, 2D spectra were iteratively analyzed using SPARKY software [55,56]. Chemical shifts assignment was obtained using the standard approach described by Wuthrich [57]. Diffusion constants of peptide over time were acquired by pseudo 2D diffusion ordered spectroscopy (DOSY) experiments by a stimulated echo bipolar pulse field gradient (stebpgp1s) program [71,72]. A total of 32 spectra with gradient strengths ranging from 2% to 98% of the maximum value were recorded. A diffusion time ∆ of 60 ms and gradient length δ of 1.0 ms were used in all the experiments. The spectra were analyzed using TopSpin Dynamics Center (Bruker, Fällanden, Switzerland). The diffusion values were obtained by fitting the peak intensity decays using the Stejskal-Tanner equation [73]:

$$f\left(g\right)=I_{0}e^{-\gamma^{2}g^{2}\delta^{2}\left(\Delta -\frac{\delta }{3}\right)D}$$

Using the Wilkins equation, it was possible to determine the hydrodynamic radius (R_h_) of Aβ(25−35) peptide from the diffusion values. We added 1,4-dioxane to a final concentration of 10 mM as internal standard. Because the hydrodynamic radius value of 1,4-dioxane is tabulated as 2.12 Å, it was used as an internal reference and used for the calculation of R_h_ [51,74]:

$$R_{h,\mathrm{prot}}=\frac{D_{\mathrm{ref}}\cdot R_{h,\mathrm{ref}}}{D_{\mathrm{prot}}}$$

where D_ref_ and R_h,ref_, respectively, are the diffusion and the hydrodynamic radius of the internal reference, and D_prot_ and R_h,prot_, respectively, are the diffusion and the hydrodynamic radius of Aβ(25−35) peptide. 

#### 4.3.2. Structure Calculations

NOESY peaks were integrated using the Gaussian fit integration method of SPARKY software. Peak volumes deriving from the assignment were translated into upper distance bounds with the CALIBA routine from the CYANA 2.1 software package [58]. Redundant and duplicate constraints were discarded for each sample, and the final list of constraints was used to generate a set of 50 structures using the CYANA protocol of simulated annealing in torsion angle space (50000 steps). Entries presenting the lowest target function value (2–12) and irrelevant residual violation (maximum violation 0.71 Å) were analyzed using Schrodinger’s Maestro 12.5.139 [75]. Procheck-NMR was used to assess the quality of the structures and to analyze the dihedral angles [59]. 

## Figures and Tables

**Table 1:** Diffusion (D) values (m^2^/s) of Aβ(25−35) in SDS obtained by DOSY experiments.

	D (m^2^/s) SDS	D (m^2^/s) Aβ(25−35)
Day 0	6.98 ± 0.06 × 10^−11^	6.78 ± 0.07 × 10^−11^
Day 4	6.67 ± 0.40 × 10^−11^	6.45 ± 0.17 × 10^−11^
Day 7	6.45± 0.04 × 10^−11^	6.43 ± 0.19 × 10^−11^
Day 14	6.58 ± 0.19 × 10^−11^	6.60 ± 0.14 × 10^−11^

**Table 2:** Statistics for the structural calculation of the NMR ensemble of Aβ(25−35) peptide at 0, 4, 7, and 14 days in SDS micelles.

	Day 0	Day 4	Day 7	Day 14
Number of Experimental Restraints after CYANA				
Total NOEs	169	203	217	217
Intra residual	112	118	123	121
Short-range	53	56	60	60
Medium-range	4	29	34	36
Long-range	0	0	0	0
RMSD				
bb/heavy Å	2.15/3.09	0.63/1.21	0.58/1.21	0.25/0.94
Ramachandran analysis				
Favorable regions	40.0%	40.6%	41.7%	84.3%
Additional allowed regions	41.7%	43.1%	29.7%	14.3%
Generously allowed regions	18.0%	14.9%	28.6%	1.1%
Disallowed regions	0.3%	1.4%	0.0%	0.3%

## Data Availability

Not applicable.

## Supplementary Materials

The following supporting information can be downloaded at: https://www.mdpi.com/article/10.3390/ijms24020971/s1.
