# Supplementary material for: Monitoring the Conformational Changes of the Aβ(25−35) Peptide in SDS Micelles: A Matter of Time
Source: Int J Mol Sci. 2023 Jan 4;24(2):971. doi: 10.3390/ijms24020971 (PMC9867351; doi:10.3390/ijms24020971)
Supplement: Supplementary file 1 [file ijms-24-00971-s001.zip › ijms-2108266-supplementary.pdf]

Table of Contents:

Figure S1: Pseudo 2D DOSY of Aβ(25-35) in SDS with 1,4-dioxane as a reference recorded at day 0 and here reported as example.

Figure S2: Intensities of Aβ(25-35) (continous line) and SDS (dashed line) signals plotted in function of the gradient strength as recorded from DOSY experiments at days 0, 4, 7 and 14.

Figure S3: 1D ^{1}H spectra of Aβ(25-35) recorded at days 0 (black), 4 (red), 7 (green) and 14 (blue) on Bruker 600 MHz in in SDS micelles.

Figure S4: High-field region (0-4.4 ppm) of 1D ^{1}H spectra of Aβ(25-35) recorded at days 0 (black), 4 (red), 7 (green) and 14 (blue) on Bruker 600 MHz in in SDS micelles.

Figure S5: Low-field region (6-8.7 ppm) of 1D ^{1}H spectra of Aβ(25-35) recorded at days 0 (black), 4 (red), 7 (green) and 14 (blue) on Bruker 600 MHz in in SDS micelles.

Figure S6: Low-field strips (7.4-8.8 ppm) of 2D-NOESY spectra of Aβ(25-35) recorded at days 0, 4, 7 and 14 on Bruker 600 MHz in in SDS micelles.

Table S1: ^{1}H Chemical Shifts of Aβ(25-35) at day 0 acquired on Bruker 600 MHz in in SDS micelles. The final pH was 7.4. NMR experiments were recorded at 25 °C.

Table S2: ^{1}H Chemical Shifts of Aβ(25-35) at day 4 acquired on Bruker 600 MHz in in SDS micelles. The final pH was 7.4. NMR experiments were recorded at 25 °C.

Table S3: ^{1}H Chemical Shifts of Aβ(25-35) at day 7 acquired on Bruker 600 MHz in in SDS micelles. The final pH was 7.4. NMR experiments were recorded at 25 °C.

Table S4: ^{1}H Chemical Shifts of Aβ(25-35) at day 14 acquired on Bruker 600 MHz in in SDS micelles. The final pH was 7.4. NMR experiments were recorded at 25 °C.

Figure S7: Ramachandran plot for each residue in the ensemble (50 models) of Aβ(25-35) structures calculated from NMR 2D spectra in SDS (A-D) and in the ensemble (10 models) of Aβ(1-40) in SDS as in PDB ID: 1BA4 (E).

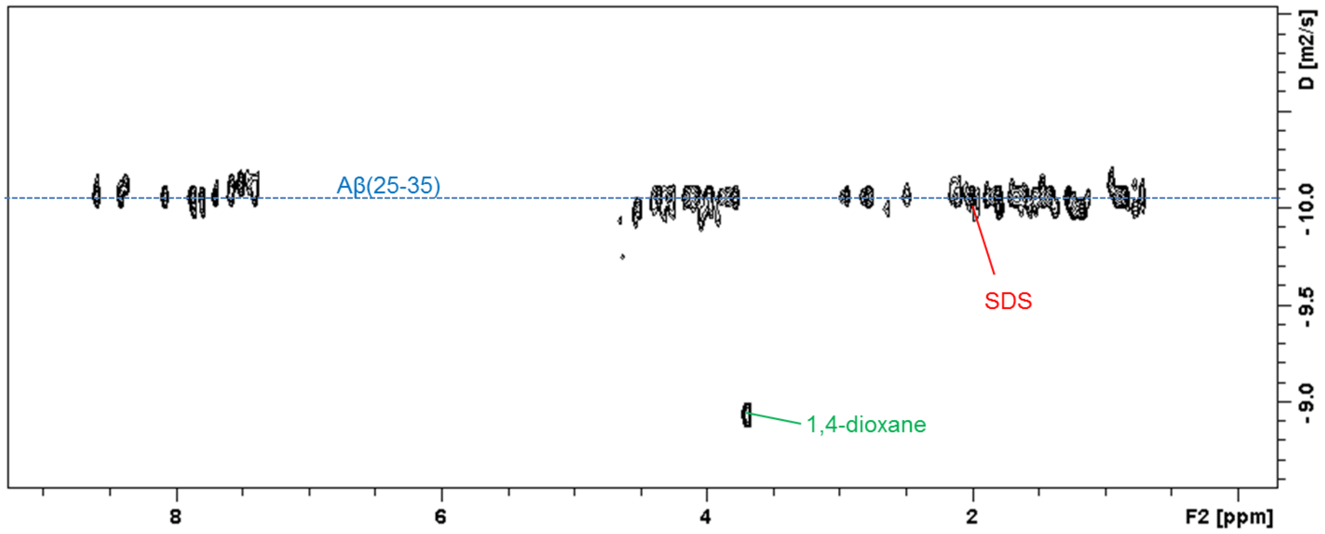

Figure S1: Pseudo 2D DOSY of Aβ(25-35) in SDS with 1,4-dioxane as a reference recorded at day 0 and here reported as example.

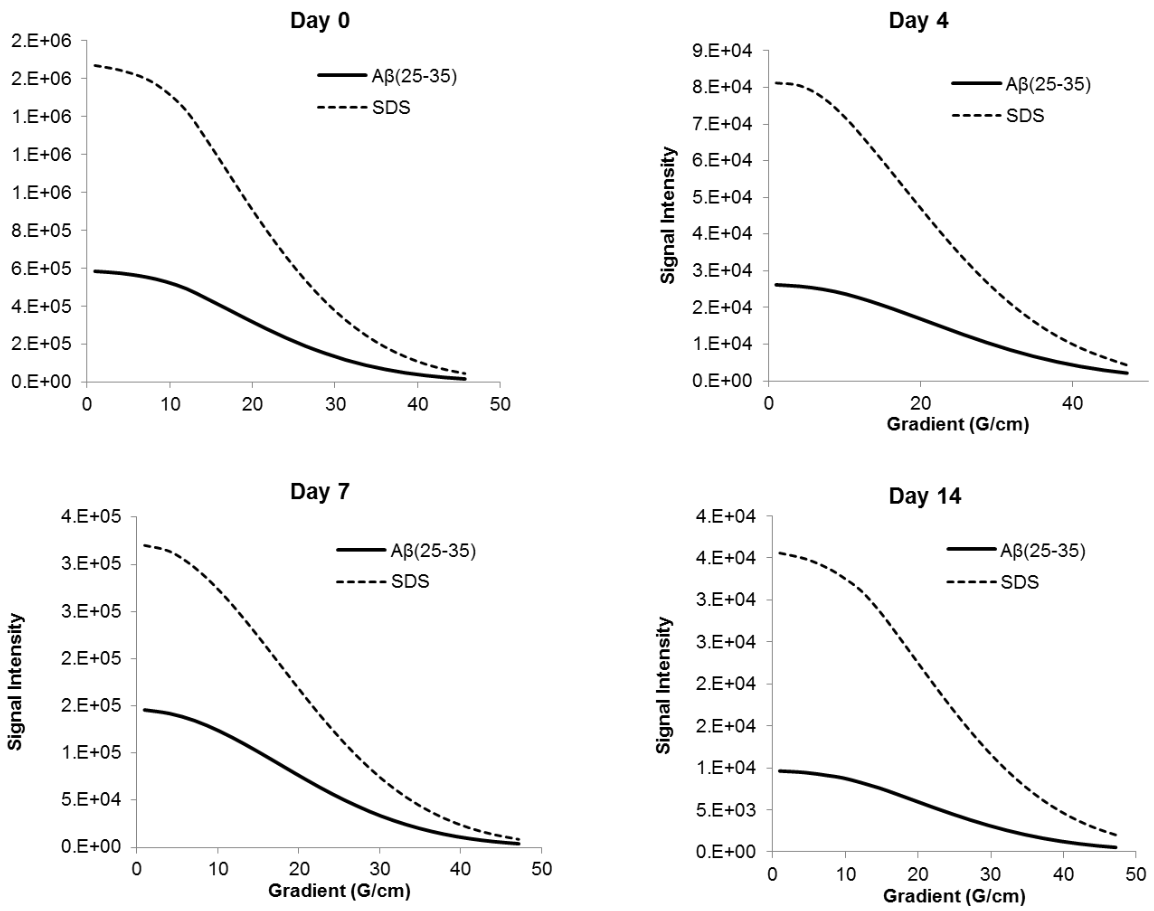

Figure S2: Intensities of Aβ(25-35) (continous line) and SDS (dashed line) signals plotted in function of the gradient strength as recorded from DOSY experiments at days 0, 4, 7 and 14.

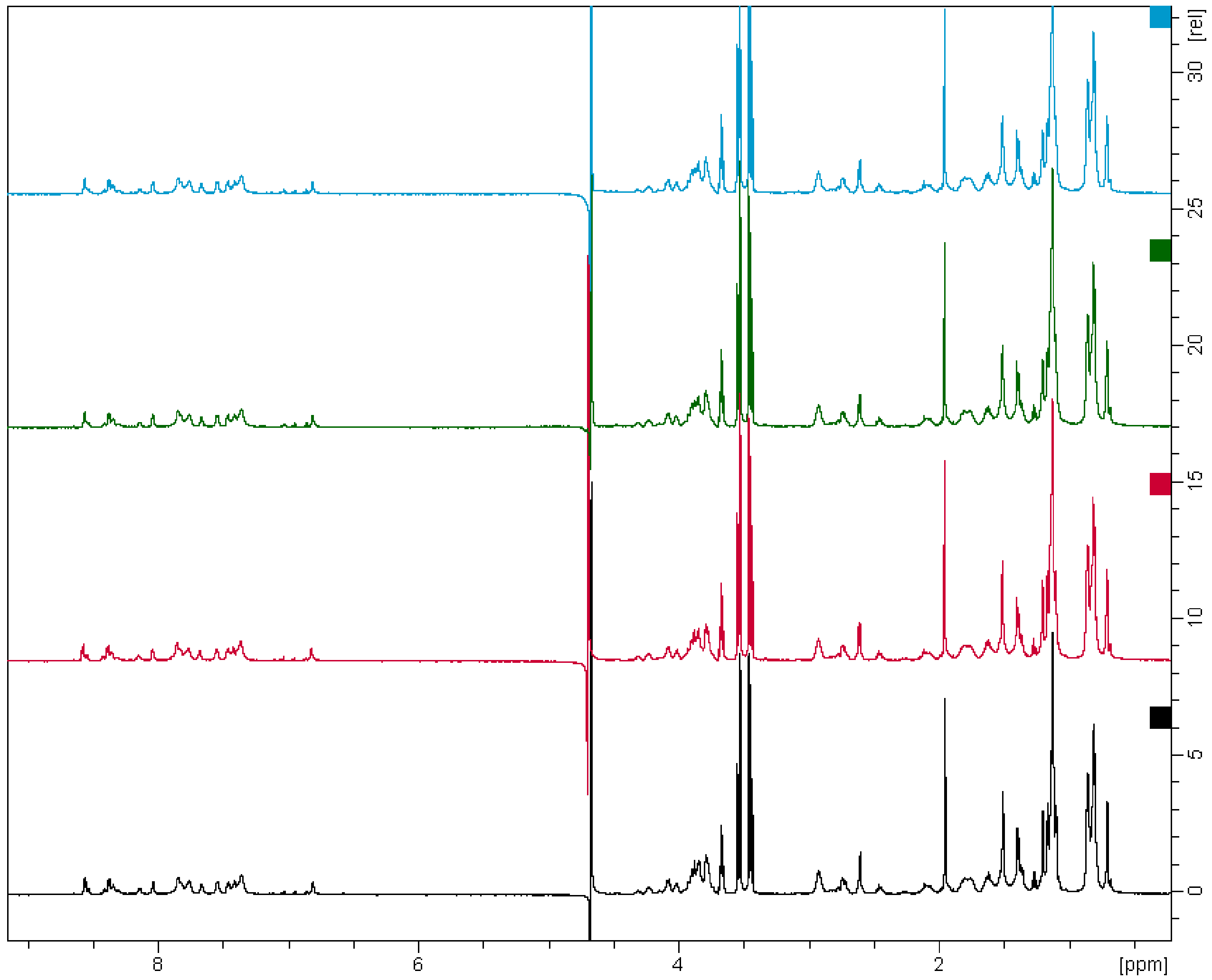

Figure S3: 1D ^{1}H spectra of Aβ(25-35) recorded at days 0 (black), 4 (red), 7 (green) and 14 (blue) on Bruker 600 MHz in in SDS micelles.

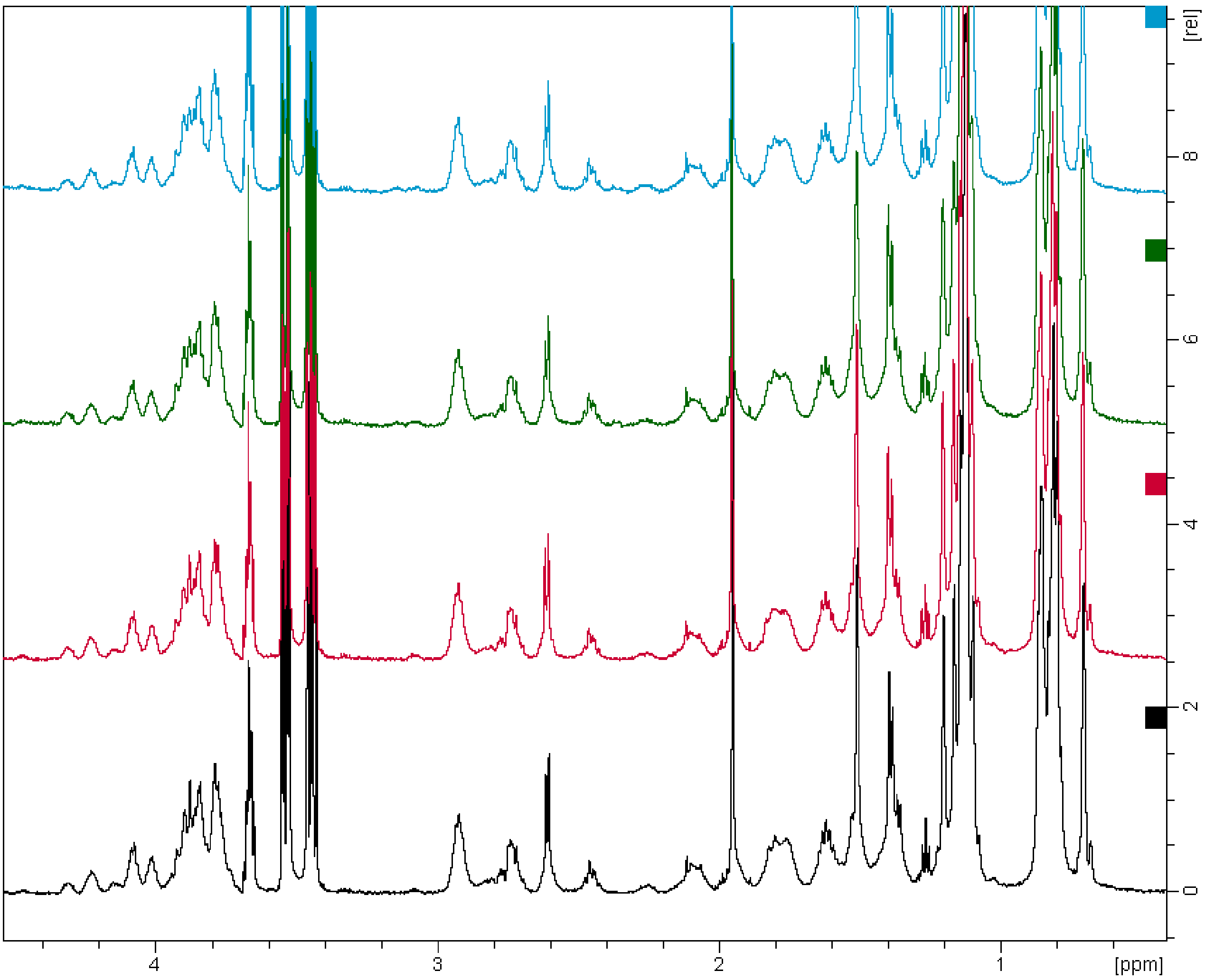

Figure S4: High-field region (0-4.4 ppm) of 1D ^{1}H spectra of Aβ(25-35) recorded at days 0 (black), 4 (red), 7 (green) and 14 (blue) on Bruker 600 MHz in in SDS micelles.

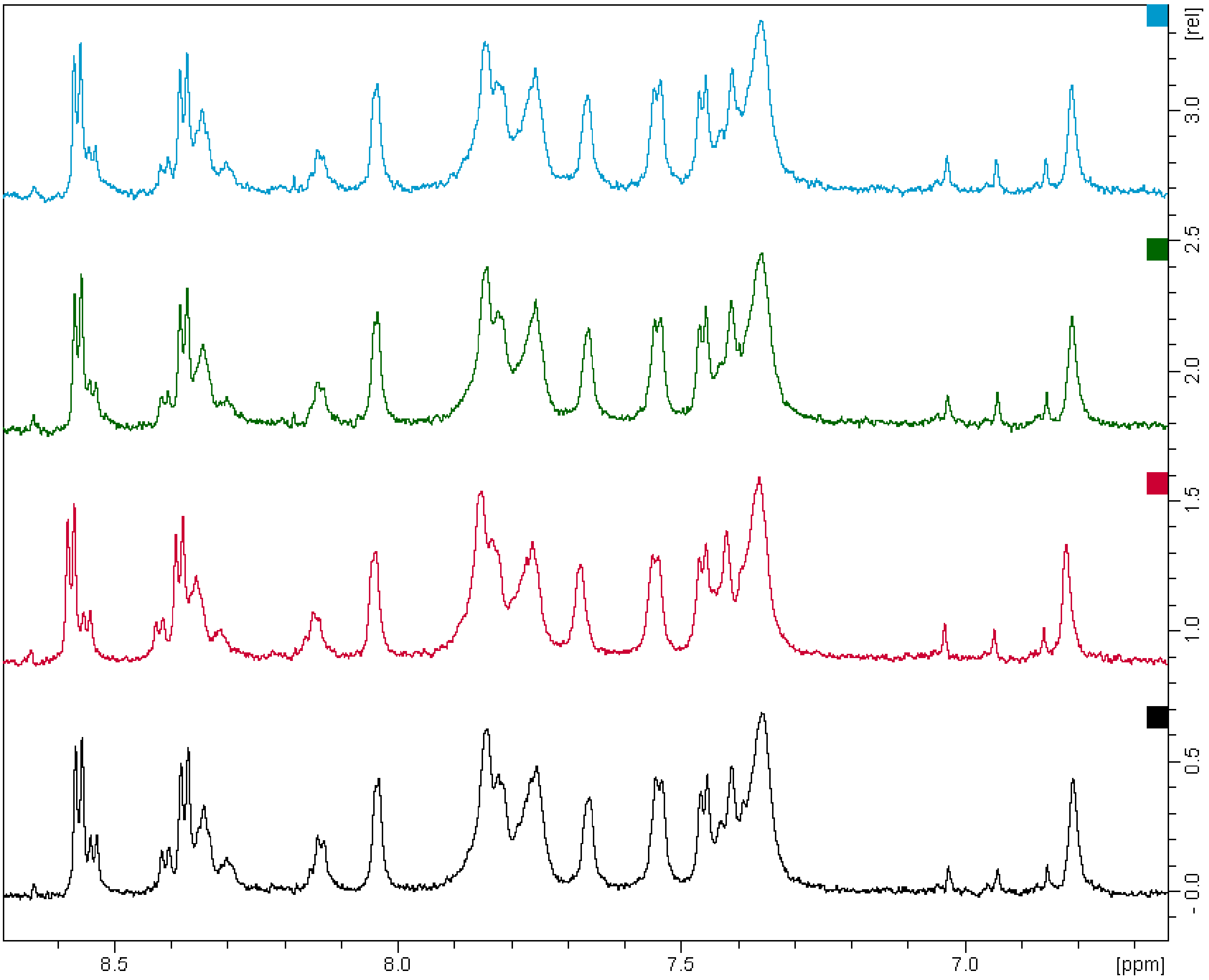

Figure S5: Low-field region (6-8.7 ppm) of 1D ^{1}H spectra of Aβ(25-35) recorded at days 0 (black), 4 (red), 7 (green) and 14 (blue) on Bruker 600 MHz in in SDS micelles.

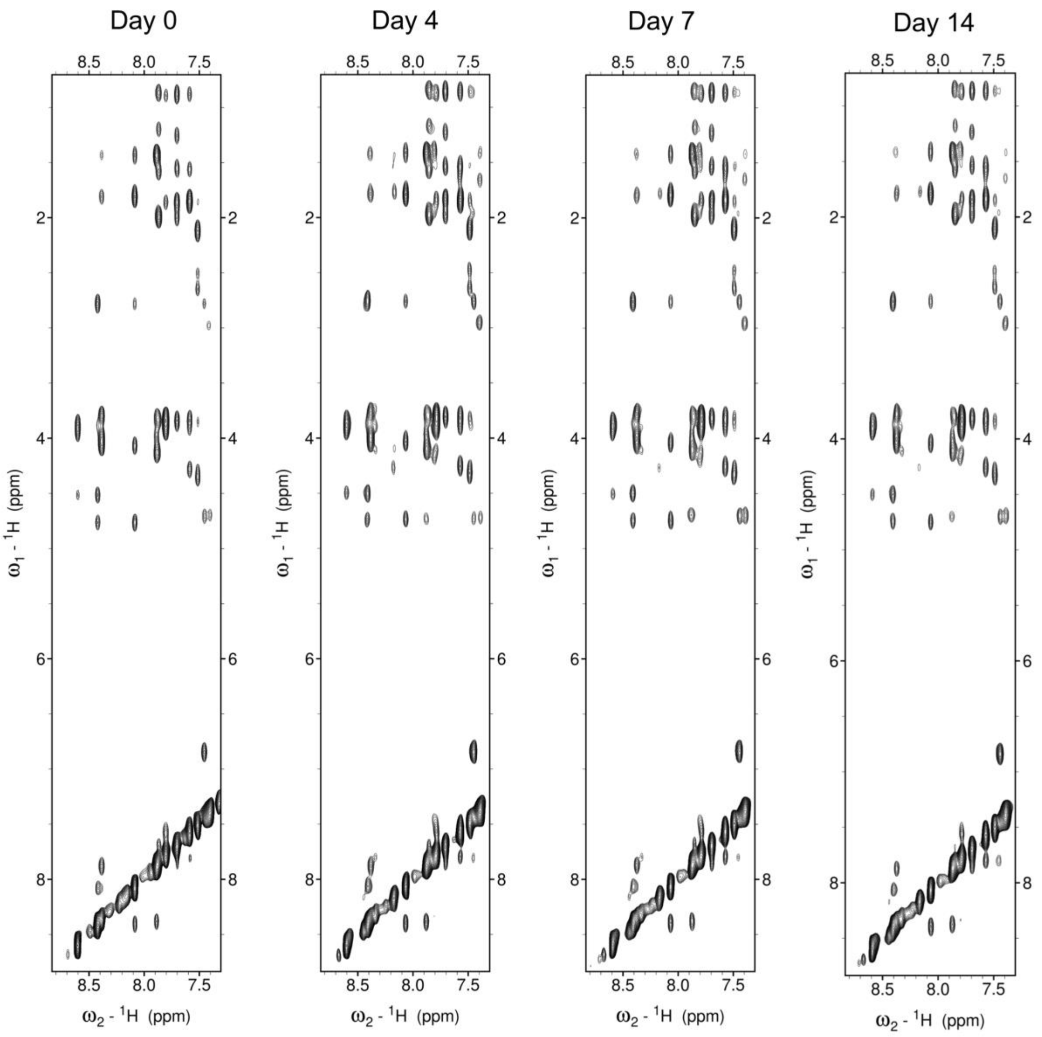

Figure S6: Low-field strips (7.4-8.8 ppm) of 2D-NOESY spectra of Aβ(25-35) recorded at days 0, 4, 7 and 14 on Bruker 600 MHz in in SDS micelles.

Table S1: ^{1}H Chemical Shifts of Aβ(25-35) at day 0 acquired on Bruker 600 MHz in in SDS micelles. The final pH was 7.4. NMR experiments were recorded at 25 °C.

Residue	HN	HA	HB	HG	HD	HE
^{25}Gly	7.89	3.96<br>3.91				
^{26}Ser	8.60	4.51	3.88<br>3.84			
^{27}Asn	8.42	4.76	2.78			
^{28}Lys	8.09	4.07	1.81	1.45<br>1.41	1.69	2.97
^{29}Gly	8.38	4.01<br>3.80				
^{30}Ala	7.88	4.13	1.44			
^{31}Ile	7.87	3.84	1.99	1.58<br>1.20	0.84<br>0.89	
^{32}Ile	7.70	3.84	1.86	1.55<br>1.25	0.86<br>0.91	
^{33}Gly	7.80	3.92<br>3.83				
^{34}Leu	7.59	4.28	1.85	1.56	0.90<br>0.85	
^{35}Met	7.51	4.37	2.13	2.65<br>2.50		

Table S2: ^{1}H Chemical Shifts of Aβ(25-35) at day 4 acquired on Bruker 600 MHz in in SDS micelles. The final pH was 7.4. NMR experiments were recorded at 25 °C.

Residue	HN	HA	HB	HG	HD	HE
^{25}Gly	7.89	3.94<br>3.90				
^{26}Ser	8.62	4.51	3.88<br>3.83			
^{27}Asn	8.43	4.75	2.77			
^{28}Lys	8.08	4.04	1.80	1.44<br>1.39	1.66	2.96
^{29}Gly	8.40	3.98<br>3.79				
^{30}Ala	7.89	4.12	1.42			
^{31}Ile	7.87	3.82	1.98	1.55<br>1.17	0.85<br>0.89	
^{32}Ile	7.72	3.82	1.84	1.55<br>1.25	0.85<br>0.89	
^{33}Gly	7.81	3.89<br>3.82				
^{34}Leu	7.59	4.27	1.84	1.55	0.89<br>0.84	
^{35}Met	7.51	4.34	2.12	2.64<br>2.49		

Table S3: ^{1}H Chemical Shifts of Aβ(25-35) at day 7 acquired on Bruker 600 MHz in in SDS micelles. The final pH was 7.4. NMR experiments were recorded at 25 °C.

Residue	HN	HA	HB	HG	HD	HE
^{25}Gly	7.87	3.91<br>3.88				
^{26}Ser	8.59	4.50	3.87<br>3.82			
^{27}Asn	8.41	4.74	2.76			
^{28}Lys	8.07	4.05	1.79	1.42<br>1.39	1.67	2.96
^{29}Gly	8.37	3.97<br>3.78				
^{30}Ala	7.87	4.11	1.42			
^{31}Ile	7.85	3.82	1.98	1.56<br>1.17	0.85<br>0.88	
^{32}Ile	7.69	3.82	1.84	1.54<br>1.24	0.84<br>0.89	
^{33}Gly	7.79	3.89<br>3.79				
^{34}Leu	7.57	4.26	1.84	1.54	0.90<br>0.84	
^{35}Met	7.49	4.34	2.11	2.64<br>2.48		

Table S4: ^{1}H Chemical Shifts of Aβ(25-35) at day 14 acquired on Bruker 600 MHz in in SDS micelles. The final pH was 7.4. NMR experiments were recorded at 25 °C.

Residue	HN	HA	HB	HG	HD	HE
^{25}Gly	7.86	3.92<br>3.88				
^{26}Ser	8.59	4.50	3.88<br>3.82			
^{27}Asn	8.41	4.74	2.76			
^{28}Lys	8.07	4.04	1.79	1.44<br>1.39	1.67	2.96
^{29}Gly	8.38	3.97<br>3.79				
^{30}Ala	7.87	4.11	1.42			
^{31}Ile	7.85	3.82	1.98	1.57<br>1.17	0.84<br>0.89	
^{32}Ile	7.70	3.82	1.84	1.54<br>1.24	0.84<br>0.88	
^{33}Gly	7.79	3.90<br>3.80				
^{34}Leu	7.57	4.26	1.84	1.55	0.89<br>0.84	
^{35}Met	7.49	4.34	2.11	2.63<br>2.48		

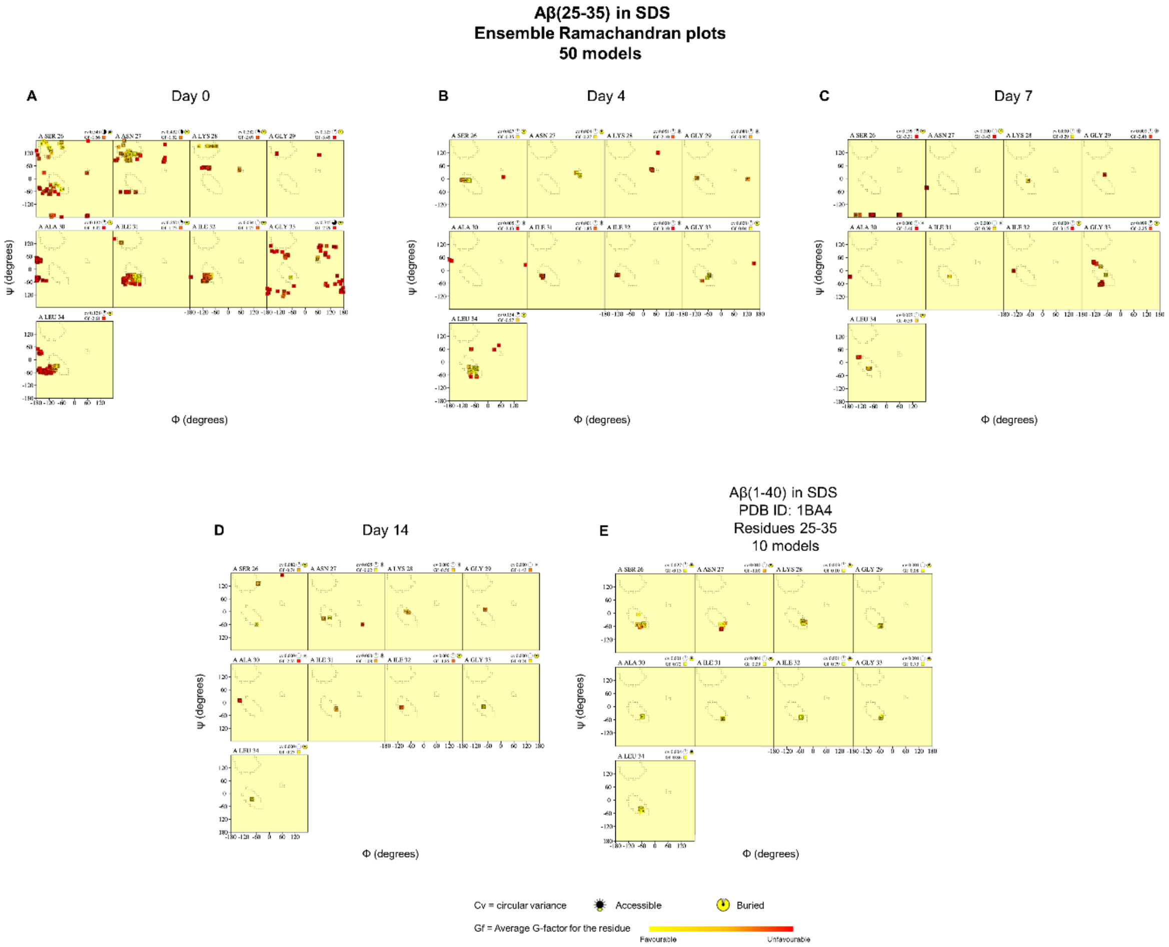

Figure S7: Ramachandran plot for each residue in the ensemble (50 models) of Aβ(25-35) structures calculated from NMR 2D spectra in SDS (A-D) and in the ensemble (10 models) of Aβ(1-40) in SDS as in PDB ID: 1BA4 (E).
